# Mitochondrial and bioenergetic dysfunction in trauma-induced painful peripheral neuropathy

**DOI:** 10.1186/s12990-015-0057-7

**Published:** 2015-09-17

**Authors:** Tony K. Y. Lim, Malena B. Rone, Seunghwan Lee, Jack P. Antel, Ji Zhang

**Affiliations:** Neurology and Neurosurgery, McGill University, Montreal, QC H3A 2B4 Canada; Alan Edwards Centre for Research on Pain, McGill University, 740 Docteur Penfield Ave, Suite 3200, Montreal, QC H3A 0G1 Canada; Faculty of Dentistry, McGill University, Montreal, QC H3A 0C7 Canada

**Keywords:** Bioenergetics, Mitochondria, Nerve injury, Neuropathic pain

## Abstract

**Background:**

Mitochondrial dysfunction is observed in various neuropathic pain phenotypes, such as chemotherapy induced neuropathy, diabetic neuropathy, HIV-associated neuropathy, and in Charcot-Marie-Tooth neuropathy. To investigate whether mitochondrial dysfunction is present in trauma-induced painful mononeuropathy, a time-course of mitochondrial function and bioenergetics was characterized in the mouse partial sciatic nerve ligation model.

**Results:**

Traumatic nerve injury induces increased metabolic indices of the nerve, resulting in increased oxygen consumption and increased glycolysis. Increased metabolic needs of the nerve are concomitant with bioenergetic and mitochondrial dysfunction. Mitochondrial dysfunction is characterized by reduced ATP synthase activity, reduced electron transport chain activity, and increased futile proton cycling. Bioenergetic dysfunction is characterized by reduced glycolytic reserve, reduced glycolytic capacity, and increased non-glycolytic acidification.

**Conclusion:**

Traumatic peripheral nerve injury induces persistent mitochondrial and bioenergetic dysfunction which implies that pharmacological agents which seek to normalize mitochondrial and bioenergetic dysfunction could be expected to be beneficial for pain treatment. Increases in both glycolytic acidification and non-glycolytic acidification suggest that pH sensitive drugs which preferentially act on acidic tissue will have the ability to preferential act on injured nerves without affecting healthy tissues.

## Background

There is accumulating evidence that mitochondrial dysfunction plays a role in conditions of painful peripheral neuropathy [1, 2]. For example, mitochondrial dysfunction has been observed in models of chemotherapy induced neuropathy [3], diabetic neuropathy [4], and in HIV-associated sensory neuropathy [5]. Furthermore, in humans, mutations in mitochondrial genes frequently result in the development of painful peripheral neuropathy, as seen in patients with Charcot-Marie-Tooth disease [6]. Clearly, mitochondria are mechanistically involved in neuropathic pain, and a further understanding and characterization of the role that mitochondria play in pain pathogenesis is required.

Under normal physiological conditions, mitochondria are responsible for the production of the majority of ATP in neurons [7]. Mitochondria produce ATP by oxidation of pyruvate through the oxidative phosphorylation respiratory chain complex. Under conditions of mitochondrial dysfunction, a lack of ATP can lead to a failure in the Na^+^/K^+^ ATPase, and in primary sensory neurons this may contribute to ectopic activity characteristic of neuropathic pain [8]. Mitochondria also have roles in the production and modulation of reactive oxygen species (ROS) [9], as well as in the maintenance of cytosolic Ca2^+^ levels [10]. Mitochondrial dysfunction leads to increased ROS and cytosolic Ca^2+^ imbalances—mechanisms which have both been previously implicated in neuropathic pain pathogenesis [11, 12]. Furthermore, a lack of ATP shifts cellular ATP production to glycolysis [13], which can result in lactate acidosis. Tissue acidosis is well recognized mechanism that can cause constant ongoing pain [14].

While there is mounting evidence to suggest that dysfunctional mitochondria play a role in peripheral neuropathy, whether mitochondrial dysfunction in peripheral tissue contributes to pain pathogenesis in trauma-induced peripheral mononeuropathy is not known. Furthermore, our knowledge of the role that cellular bioenergetics play in neuropathic pain is unknown. The goal of this manuscript was to fill this knowledge gap by characterizing the time course and chronicity of mitochondrial and bioenergetic dysfunction in a model of painful trauma-induced peripheral neuropathy. Partial sciatic nerve ligation (PSNL) decreases the paw withdrawal thresholds shortly after the surgery and this mechanical hypersensitivity persists for at least 1 month [15]. This work demonstrates that traumatic peripheral nerve injury, e.g., PSNL, induces persistent mitochondrial and bioenergetic dysfunction, and suggests that these mechanisms contribute to pain pathogenesis and can be exploited to develop pharmaceutical agents which act preferentially at injured nerves.

## Results

### The bioenergetic profile of mouse sciatic nerves can be measured using metabolic poisons

**Fig. 1:**
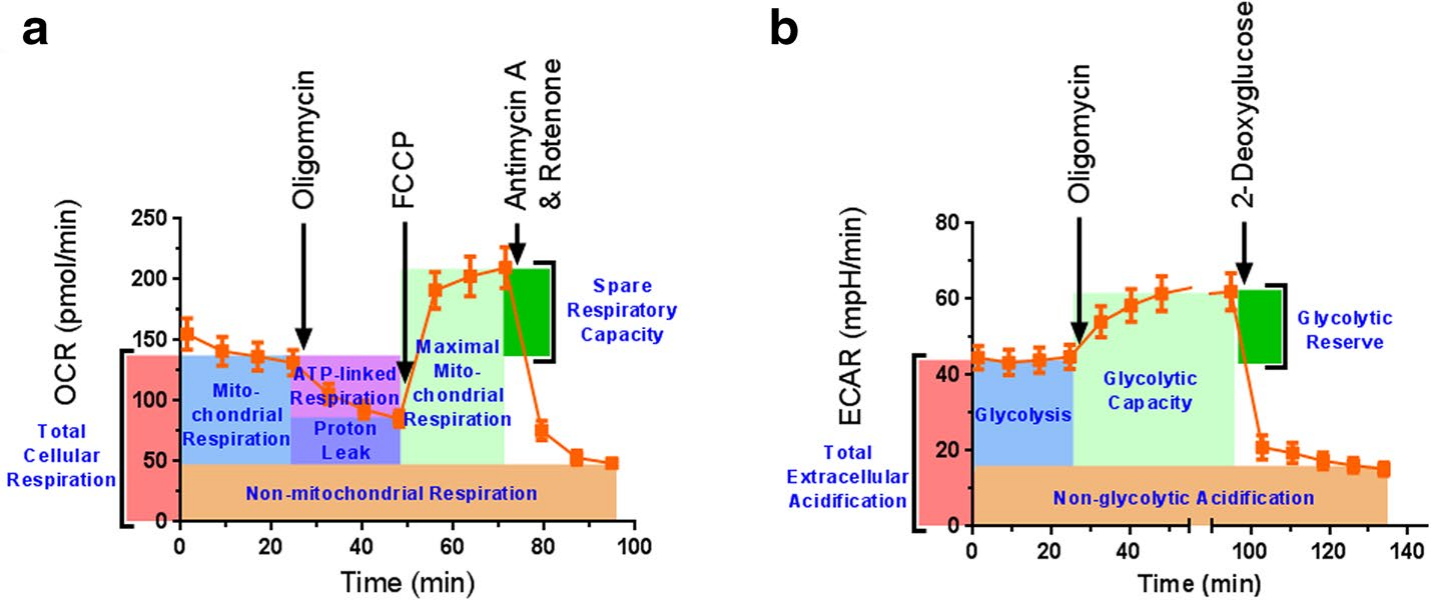
The bioenergetic profile of mouse sciatic nerves can be assessed by Seahorse metabolic assay. Sciatic nerves from mice were isolated and cut into small 1 mm hemi-segments. Tissue from a 3 mm long segment of nerve was placed into a single well. **a** Oxygen consumption rate was measured, and the oxygen consumption rate in response to oligomycin, FCCP, and antimycin A with rotenone was determined. This allowed ex vivo measurement of oxygen consumption specific to total cellular respiration, mitochondrial respiration, mitochondrial ATP production, mitochondrial proton leak, mitochondrial maximal respiration, mitochondrial spare respiratory capacity, and non-mitochondrial respiration. **b** Extracellular acidification rate can also be measured by this method. Ex vivo measurement of the extracellular acidification rate corresponding to total extracellular acidification, glycolysis, glycolytic capacity, glycolytic reserve, and non-glycolytic acidification can be measured following administration of oligomycin and 2-deoxyglucose to the tissue culture medium

A method to examine the bioenergetics profile of mice nerves was developed. Oxygen consumption and extracellular acidification rates from mouse sciatic nerves ex vivo were measured with the Seahorse XF extracellular flux analyzer. Oligomycin, FCCP, and antimycin A with rotenone was used to measure oxygen consumption linked to: total respiration, ATP-linked respiration, proton leak, non-mitochondrial respiration, maximal mitochondrial respiration, and the spare respiratory capacity (Fig. 1a). Oligomycin and 2-deoxy-d-glucose were used to measure extracellular acidification linked to: glycolysis, non-glycolygic acidification, glycolytic capacity, and glycolytic reserve (Fig. 1b).

### Nerve injury causes a persistent increase in total oxygen consumption and extracellular acid production

**Fig. 2:**
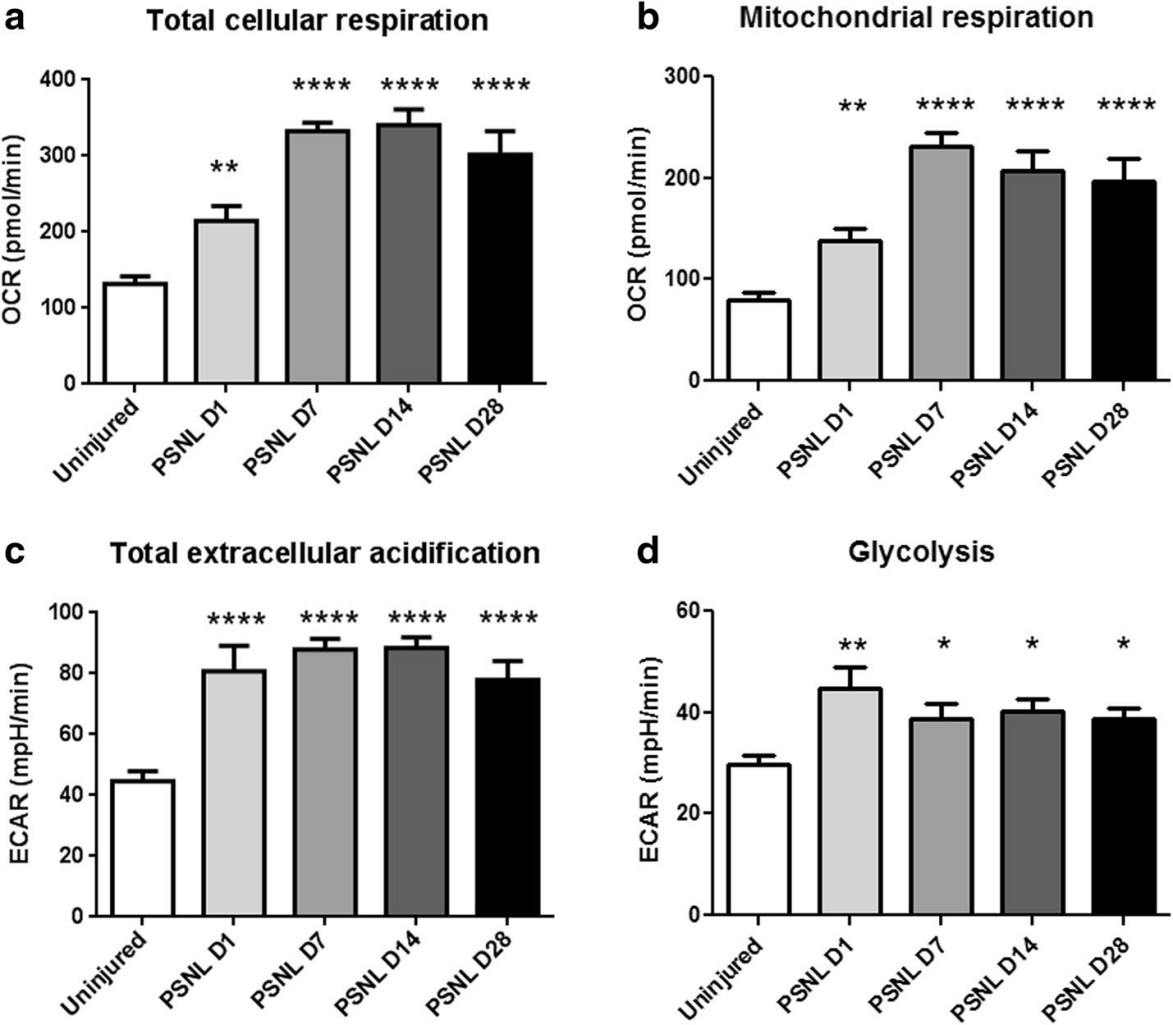
Partial sciatic nerve ligation causes persistently increased metabolic needs of injured nerves. **a** Total cellular oxygen consumption is persistently increased following nerve injury. This demonstrates that under ex vivo conditionsf **b** The oxygen consumption specific to mitochondrial respiration is also persistently increased following nerve injury. **c** Total extracellular acidification levels are persistently increased following nerve injury, demonstrating that injured nerves produce more acid than uninjured nerves. **d** Glycolysis specific extracellular acidification is also increased

Following partial sciatic nerve ligation, as soon as 1 day post-surgery, total oxygen consumption levels of nerves were persistently increased (Fig. 2a). In a similar manner, mitochondrial respiration of nerves was also increased post-injury (Fig. 2b). Nerve injury persistently increased total extracellular rates of acidification as soon as 1 day post-injury (Fig. 2c). Glycolysis specific extracellular acidification was significantly increased as well (Fig. 2d).

### Mitochondria in injured nerves have reduced ATP-linked respiration, spare capacity, maximal mitochondrial respiration, and increased proton leak

**Fig. 3:**
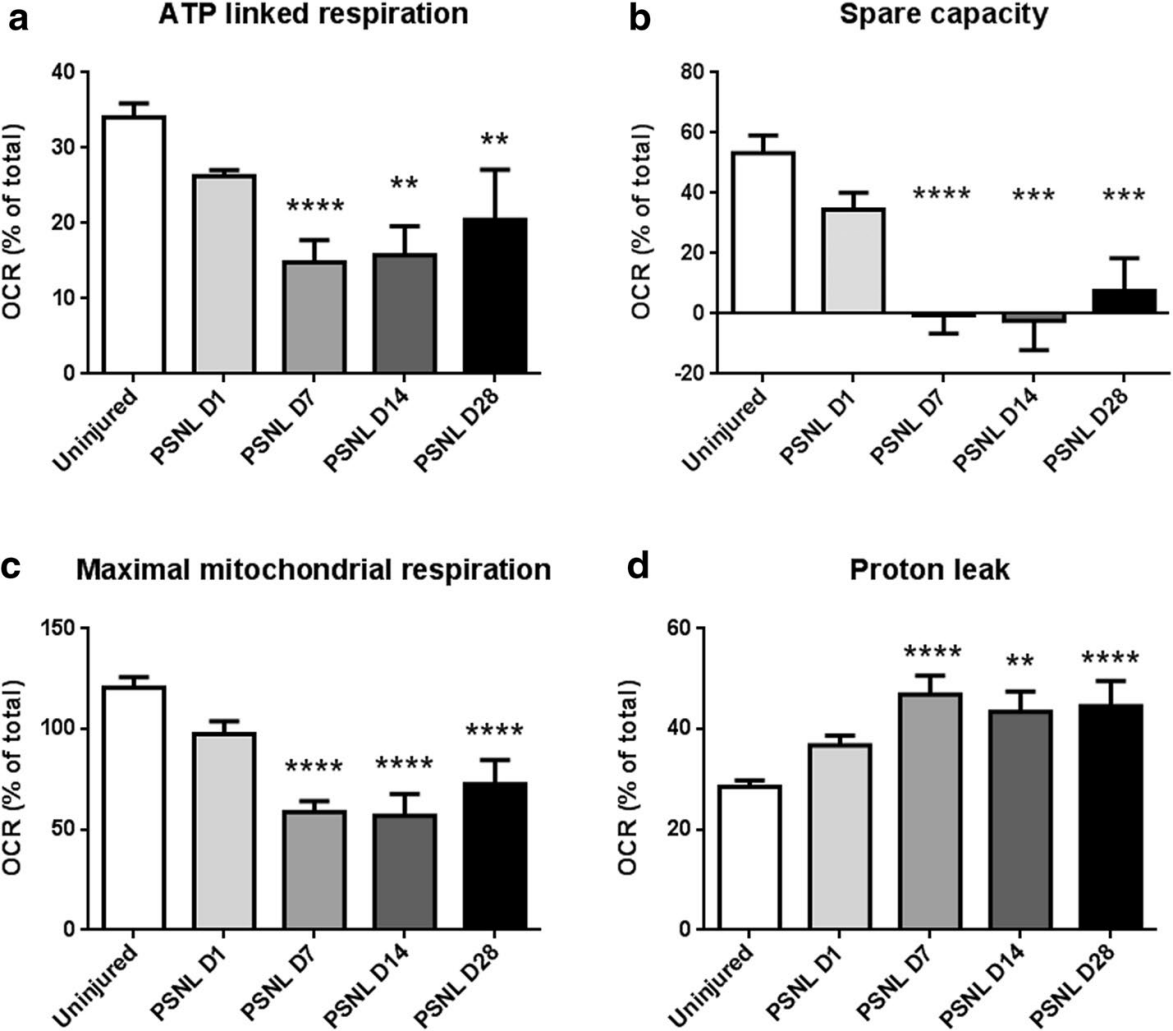
Partial sciatic nerve ligation induces persistent mitochondrial dysfunction in injured nerves. **a** The proportion of the oxygen consumption rate specific to mitochondrial ATP production is persistently reduced following partial sciatic nerve ligation, demonstrating dysfunction in mitochondrial ATP production. **b** Additionally, the mitochondria of injured nerves have no spare capacity. When stimulated pharmacologically with a proton uncoupling agent, mitochondria are unable to consume any more oxygen after nerve injury. This suggests that the mitochondria in injured nerves are under bioenergetic stress and are already operating at their maximum capabilities for ATP production. **c** Maximal mitochondrial respiration is persistently reduced following nerve injury. This demonstrates that nerve injury reduces the ability of mitochondria to perform oxidative processes. **d** Futile proton cycling, or proton leak, is increased in mitochondria following nerve injury. Mitochondria undergo changes to allow more protons to passively dissipate the proton motive force after nerve injury. This avails fewer protons for ATP synthesis

Oligomycin inhibits ATP synthase by blocking the proton channel subunit F_0_. Thus, by adding oligomycin to the nerves, the respiration specific to ATP synthesis can be measured. Following nerve injury, oxygen consumption linked to ATP production decreased, and was significantly reduced by day 7 post-injury, until at least day 28 post-injury (Fig. 3a). FCCP is a mitochondrial uncoupling agent which discharges the pH gradient across the inner mitochondrial membrane. This induces the mitochondria to work at their maximum respiratory rate, allowing measurement of mitochondrial spare capacity and maximum mitochondrial respiration. By day 7 post-injury, mitochondria in injured nerves have significantly reduced spare capacity—at levels near zero (Fig. 3b). Spare capacity is significantly reduced until at least day 28, suggesting persistent effects of nerve injury on mitochondria. Not only is the spare capacity reduced by nerve injury, maximal mitochondrial respiration is also significantly reduced after day 7 post-injury (Fig. 3c). Rotenone and antimycin A are inhibitors of the electron transport chain at complex I and III, respectively. Subtracting oxygen consumption after oligomycin from oxygen consumption after antimycin A and rotenone administration gives the mitochondrial oxygen consumption that is unrelated to ATP synthesis, or proton leak. Proton leak is significantly increased after day 7 to at least day 28 post-injury (Fig. 3d).

### Injured nerves have reduced glycolytic reserve and glycolytic capacities

**Fig. 4:**
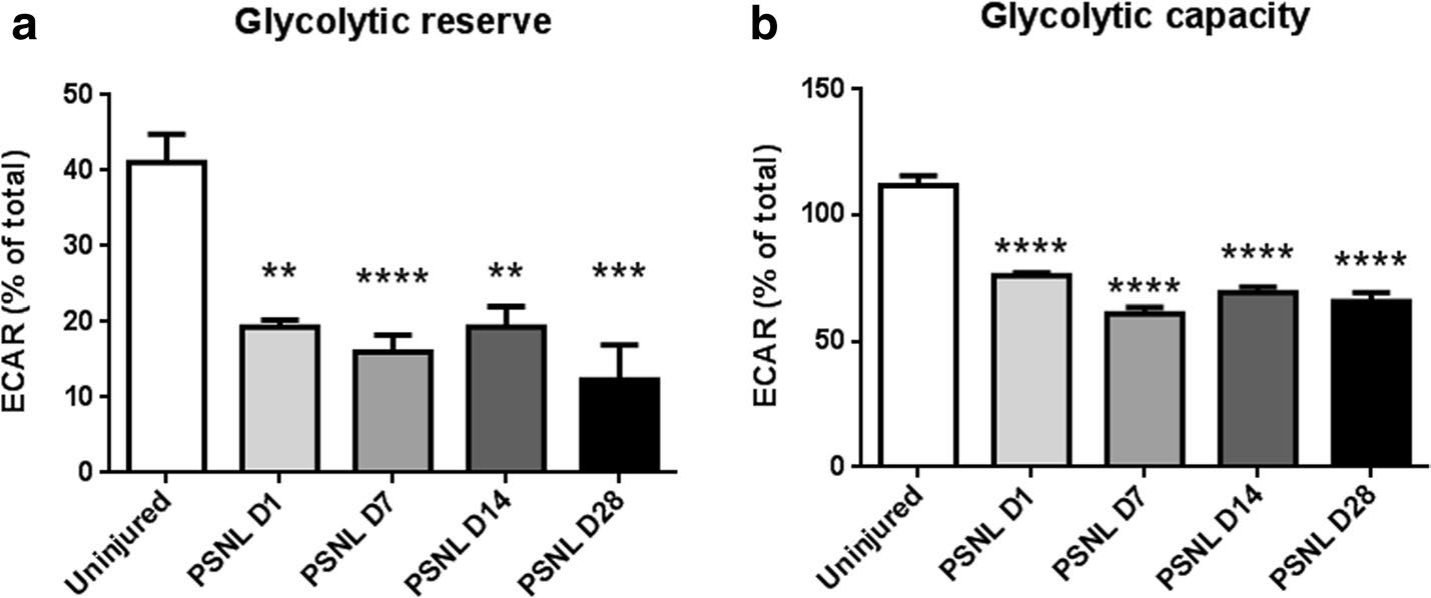
Partial sciatic nerve ligation induces persistent glycolytic dysfunction in injured nerves. **a** The proportion of the extracellular acidification rate specific to glycolysis is persistently reduced following partial sciatic nerve ligation. This demonstrates that the ability of the nerve to increase glycolytic activity in response to bioenergic stress is reduced following nerve injury. **b** Glycolytic capacity is also persistently reduced following partial sciatic nerve ligation. A reduction in glycolytic capacity suggests injured nerve tissue is less capable of producing ATP by glycolytic mechanisms

The glycolytic reserve is a measure of the ability of the tissue to increase glycolytic flux to respond to an enhanced need for ATP by glycolytic flux. This can be measured by introducing oligomycin to the tissue. The glycolytic reserve was significantly decreased after nerve injury, as early as day 1 post-injury, and persisted until at least day 28 post-injury (Fig. 4a). Glycolytic capacity measures the entire ability of the tissue to produce ATP by glycolysis. Glycolytic capacity was measured and it was also similarly significantly reduced in a persistent manner as early as day 1 post-injury (Fig. 4b).

### Nerve injury does not affect non-mitochondrial respiration, but increases non-glycolytic acidification

**Fig. 5:**
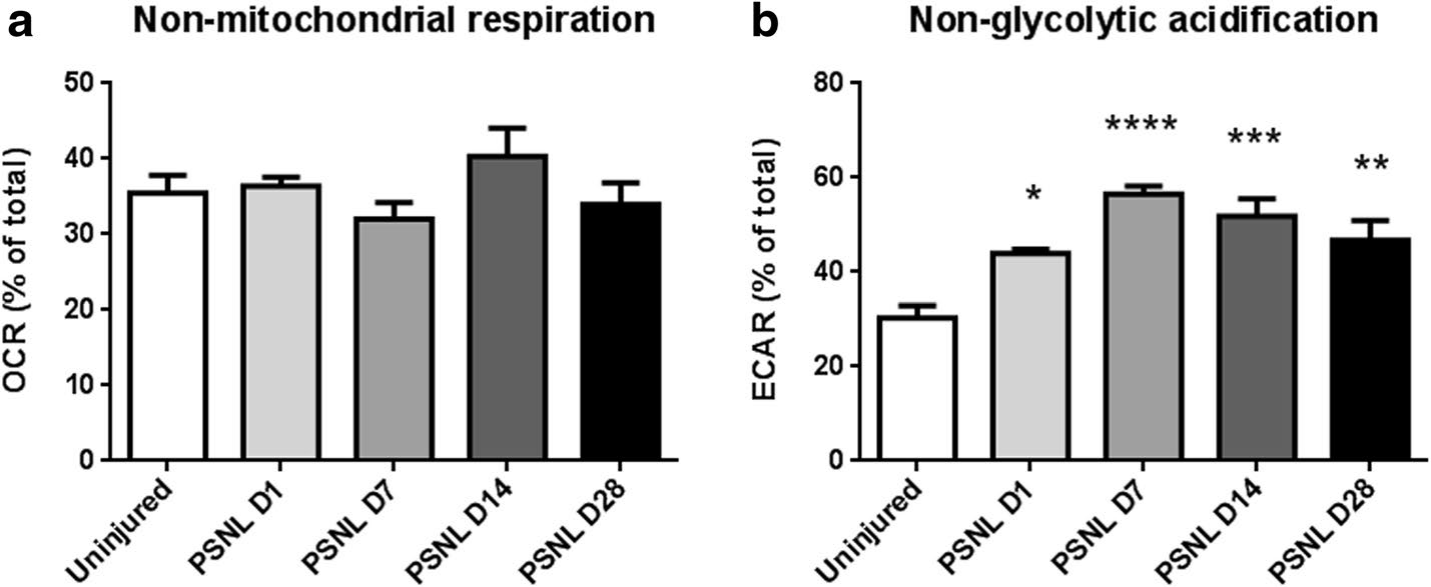
Nerve injury does not affect non-mitochondrial respiration, but increases non-glycolytic acidification. **a** Nerve injury induces no proportionate change in non-mitochondrial respiration. This suggests that oxygen consumption of non-mitochondrial processes such as cellular oxidases do not undergo alterations following nerve injury. **b** Nerve injury induces a persistent increase in non-glycolytic acidification. This suggests that non-glycolytic processes which produce acids are increased following nerve injury. Increased acidification may have undesirable effects on sensory neuron excitability

Non-mitochondrial respiration was measured with the addition of antimycin A and rotenone to the tissue. No significant changes in non-mitochondrial respiration were observed after nerve injury (Fig. 5a). In a similar fashion, non-glycolytic acidification can be measured by administering 2-deoxy-d-glucose to the tissue which inhibits glycolysis. Interestingly, non-glycolytic acidification was increased as early as 1 day after nerve injury, and the increase persisted until at least day 28 (Fig. 5b).

## Discussion

### Traumatic injury causing painful mononeuropathy increases the metabolic needs of the nerve

Following nerve injury, large numbers of immune cells enter into the nerve to contribute to neuroinflammation, Wallerian degeneration and neuropathic pain [15, 16]. An approximate 3-fold increase in the number of DAPI nucleated cells is observed [8], which agrees with the 3-fold increase in total oxygen consumption levels seen in Fig. 2a. Mitochondrial specific respiration is also increased (Fig. 2b), suggesting that increased oxygen consumption by mitochondria underlie at least a part of the increase in total oxygen consumption following nerve injury.

Similarly, Fig. 2c shows a marked increase in total extracellular acidification rates following nerve injury. Likely, one of the factors that explains the increased extracellular acidification rate is the increased number of cells within injured nerves, which results in increased lactic acidosis [8]. Indeed, extracellular acidification rates which can be attributed to glycolysis is significantly increased, as seen in Fig. 2d. This demonstrates that at least some of the increase in total acidification rates following nerve injury can be attributed to increased glycolysis.

### ATP synthase activity is repressed by traumatic nerve injury causing painful mononeuropathy

Under physiological conditions, mitochondria produce ATP through the oxidation of glucose metabolites, such as pyruvate and NADH. This is performed by the F_0_F_1_ ATP synthase which resides on the inner mitochondrial membrane. Oligomycin is an inhibitor of ATP synthase and applying it to nerve tissue allows the measurement of ATP synthase activity. Interestingly, while mitochondrial specific oxygen consumption increased as described above, the proportion of oxygen consumption specific to ATP synthase activity is reduced by nerve injury (Fig. 3a).

Hypoxia is present in injured nerves [8], and likely contributes to the inhibition of ATP synthase. A lack of oxygen slows oxidative phosphorylation, decreasing the mitochondrial membrane potential and causes the F_0_F_1_ ATP synthase to run in reverse, resulting in a dissipation of ATP [17]. To avoid this potentially disastrous situation, the F_0_F_1_ ATP synthase is regulated by the protein IF_1_, which reversibly inhibits the F_0_F_1_ ATP synthase under conditions of low pH and low mitochondrial membrane potentials, which occur during hypoxia. This mechanism likely explains why nerve injury reduces oxygen consumption attributable to ATP synthase.

### Traumatic nerve injury causing painful mononeuropathy induces severe bioenergetic stress on mitochondria

Spare respiratory capacity measures the ability of the tissue to respond to an increased ATP demand or increased workload. A failure of the mitochondria to meet ATP demands under metabolically stressful conditions can result in cell death [18] and neurodegeneration [19, 20]. After nerve injury, a reduction in the spare respiratory capacity is observed, with mitochondria in injured nerves operating with no spare capacity (Fig. 3b). Thus, traumatic nerve injury induces a profound oxidative stress on the injured tissue, and the nerve has little to no ability to increase mitochondrial activity to respond to further metabolic distress.

### Traumatic nerve injury causing painful mononeuropathy negatively affects the ability of mitochondria to perform oxidative phosphorylation

The maximal respiratory capacity is a measure of both the ability of a cell to supply substrates to mitochondria, as well as the ability of the mitochondria to oxidize the substrates. Hypoxia is in fact a regulator of oxidative phosphorylation machinery at a number of locations and can explain the reduced maximal respiratory capacity after nerve injury. Hypoxia inducible factor-1 (HIF-1) induces pyruvate dehydrogenase kinase, which in turn inhibits pyruvate dehydrogenase, limiting the amount of pyruvate available for oxidative phosphorylation [21]. Furthermore, hypoxia regulates complex IV of the electron transport chain, also known as cytochrome C oxidase (COX), and reduces the maximal rate of COX activity [22, 23]. Thus, hypoxia—which is present after nerve injury—reduces both the ability of the cell and mitochondria to carry out oxidative phosphorylation, which will decrease the maximal respiratory capacity of injured nerves (Fig. 3c).

### Traumatic injury causing painful mononeuropathy causes mitochondria to increase futile proton cycling, further contributing to mitochondrial dysfunction

As an alternative to ATP generation, the protonmotive force generated by the electron transport chain can also be consumed by leaking back across the inner mitochondrial membrane. Studies in rat have found that proton leak contributes to approximately 25 % of the resting respiratory rate [24], a value similar to the resting proton leak in mouse sciatic nerve which was determined to be approximately 28 % (Fig. 3d). After nerve injury, proton leak increases to approximately 45 % of the total respiratory rate. Thus, after nerve injury, more of the proton motive force generated by mitochondria is dissipated, resulting in even less ATP generated by mitochondria.

Furthermore, mitochondria regulate and increase proton leak in order to and reduce ROS generation [25]. Previously, it has been demonstrated that nerve injury induces ongoing and persistent vascular dysfunction, resulting in ischemia and hypoxia [8]. It is well known that mitochondria in ischemic tissue generate reactive oxygen species (ROS) [26]. Indeed, the role of increased ROS is well characterized in neuropathic pain [11]. The observation that proton leak increased after nerve injury, is likely due to mitochondria responding to an increased presence of ROS and upregulating proton leak in response.

### Reduced glycolytic reserve and capacity demonstrates persistent bioenergetic deficit after traumatic injury causing painful mononeuropathy

The glycolytic reserve provides a measure of the ability of the tissue to accommodate increased ATP needs. Following nerve injury, injured nerve has a reduced glycolytic reserve (Fig. 4a), suggesting that the tissue is under bioenergetic stress and has little ability to accommodate an increase in ATP demand. Further evidence of impaired glycolysis is the observation of a reduction in glycolytic capacity (Fig. 4b). Normally, after exposure to bioenergetic stressors such as hypoxia, cells can respond by increasing glycolysis related enzymes through HIF-1 [21, 27]. This leads to the Warburg effect, whereby cells switch their metabolic pathways, favoring glycolysis over oxidative phosphorylation [28]. However, while an overall increase in glycolysis is observed after nerve injury, glycolytic mechanisms appear to be dysfunctional. Likely, a persistent oxygen and glucose deficit due to vascular dysfunction induces ATP conservation mechanisms [29, 30]. This metabolic suppression may have functional implications on pain, resulting in a decrease in the Na^+^/K^+^ ATPase and increased nociceptor excitability [8].

### Mitochondria are the major source of reactive oxygen species following traumatic nerve injury causing painful mononeuropathy

While most oxygen is consumed by cells in pathways which involve oxidative phosphorylation, oxygen can also be consumed by non-mitochondrial activities. Examples of these processes include the NOX family of NADPH oxidases [31], and xanthine oxidases [32]. Both of these enzymes are involved in producing cellular ROS and are not involved in mitochondrial respiration. The lack of any substantial change in non-mitochondrial respiration from injured nerves (Fig. 5a) demonstrates that the source of the majority of ROS after nerve injury is the mitochondria themselves.

### Non-glycolytic pathways are a major source of protons following traumatic nerve injury causing painful mononeuropathy

Non-glycolytic acidosis is persistently increased by nerve injury (Fig. 5b). After nerve injury the tissue produces acid though mechanisms which are not involved with ATP synthesis. Enhanced tissue acidification has been described in conditions of chronic inflammation, such as asthma [33], rheumatoid arthritis [34], and arthrosclerosis [35]. These results, in conjunction with the results demonstrating increased glycolysis following nerve injury, suggest that bioenergetic dysfunction following nerve injury induces chronic acidosis of the nerve. The presence of proton sensing ion channels such as TRPV1 and ASICs in sensory neurons has been verified numerous times in the past [36–39], but whether or not these ion channels contribute to pain pathogenesis in neuropathic conditions is unclear, as little evidence of acidosis after nerve injury has been previously demonstrated. However, the results here establish that increased proton production by both glycolysis and non-glycolytic pathways occur, as early as 1 day after nerve trauma.

### Bioenergetic and mitochondrial dysfunction contribute to neuropathic pain pathophysiology

A deficit in cellular ATP is expected to result in a reduction in the activity of the Na^+^/K^+^ ATPase, which will elevate the resting membrane potential and lead to spontaneous activity of sensory neurons [1]. Furthermore, a reduction in cellular ATP levels should simulate increased glycolysis, which can result in metabolic acidosis, and activation of proton sensitive ion channels such as TRPV1 and ASICs. After nerve injury, resident and infiltrating macrophages activate and polarize to the M1 pro-inflammatory phenotype [40]. Excessive immune responses by M1 macrophages can be detrimental to the host [41] and are neurotoxic [42]. M1 macrophages mostly obtain their energy through anaerobic glycolysis [43, 44], and the presence of these macrophages likely also accounts for the increased glycolysis observed after nerve injury.

### Implications for future pharmacotherapy of neuropathic pain

Altering the metabolic programming of macrophages can alter their phenotype. For example, blocking oxidative metabolism polarizes macrophages to the M1 phenotype [45]. M1 polarized macrophages produce ROS, secrete pro-inflammatory cytokines and chemokines, contribute to lactate acidosis, and cause tissue damage [46]. Conversely, forcing oxidative metabolism in M1 macrophages polarizes the macrophage to the anti-inflammatory tissue repair M2 phenotype [45]. Thus, pharmacological agents which inhibit glycolytic metabolism in macrophages are expected to produce beneficial effects on pain by attenuating the harmful M1 macrophage phenotype.

Following traumatic nerve injury, vascular dysfunction leads to persistent hypoxia of the nerve [8]. The lack of oxygen leads to mitochondrial dysfunction, and an energy deficit within the nerve. This energy deficit will lead to a reduction in the activity of the Na^+^/K^+^ ATPase, which will elevate the resting membrane potential and lead to spontaneous activity of sensory neurons [1]. Drugs which protect mitochondria from dysfunction are expected to have beneficial effects following traumatic nerve injury. For example, previous reports have described efficacy of mitoprotective drugs such as acetyl-l-carnitine in the chronic constriction injury model of painful traumatic neuropathic pain [47]. Our work provides a clear mechanism how mitochondrial protection is beneficial for trauma-induced neuropathic pain.

In addition, our study demonstrates that after nerve injury, bioenergetic dysfunction induces persistent acidosis of the nerve. This persistent acidified state provides the rationale for the creation of drugs which have increased potency under acidic conditions, such as those which have been created for cardiac arrhythmia [48]. An ion channel type blocking drug which is more active at acidic conditions will have enhanced therapeutic ratios, as the drug would preferentially affect injured nerves without affecting healthy nervous tissue.

## Conclusions

Traumatic nerve injury induces increased oxygen consumption and glycolysis, resulting in hypoxia and acidosis. Increased metabolic needs of the nerve are concomitant with bioenergetic and mitochondrial dysfunction. Mitochondrial dysfunction is characterized by repression of ATP synthase, reduced activity of electron transport chain activity and increased futile proton cycling. Bioenergetic dysfunction is characterized by reduced glycolytic reserve, reduced glycolytic capacity, and increased non-glycolytic acidification. Pharmacological agents which seek to normalize mitochondrial and bioenergetic dysfunction are expected to be beneficial for pain treatment. Increases in both glycolytic acidification and non-glycolytic acidification suggest drugs that preferentially act on acidic tissue may have preferential activity at injured nerves without affecting healthy tissues.

## Materials and methods

### Animals

Experiments were carried out in adult C57BL/6 mice (male, 20–25 g in weight) purchased from Charles River Laboratories (Quebec, Canada). Mice were housed four to five per cage, in a temperature- and humidity-controlled vivarium, on a 12/12 h light/dark cycle beginning at 7:00 AM, with access to rodent chow and water ad libitum. All experiments were approved by the Institutional Animal Care and Use Committee of McGill University (Permit #5775) and conformed to the ethical guidelines of the International Association for the Study of Pain.

### Drugs and materials

Oligomycin A, Carbonyl cyanide 4-(trifluoromethoxy)phenylhydrazone (FCCP), rotenone, antimycin A, and 2-deoxy-d-glucose were purchased from Sigma-Aldrich. Oligomycin A and FCCP were dissolved in ethanol, antimycin A and rotenone were dissolved in DMSO before dilution. 2-deoxy-d-glucose was dissolved in supplemented XF base media and re-buffered to pH 7.4.

### Nerve injury model

Partial sciatic nerve ligation (PSNL) was performed according to the method of Seltzer et al. [49], adapted to mice [50]. Nerve injury according to this model induces a symptoms modeling neuropathic pain in humans, including persistent mechanical allodynia. Briefly, mice were anesthetized with isoflurane (3 % for induction and maintenance; Baxter Corperation) and under aseptic conditions the left sciatic nerve was exposed at high-thigh level. The dorsum of the nerve was carefully freed from surrounding connective tissues at a site near the trochanter just distal to the point at which the posterior biceps semitendinosus nerve branches off the common sciatic nerve. An 8–0 silk suture was inserted into the nerve with a 3/8 curved, reversed-cutting mini-needle, and tightly ligated so that the dorsal 1/3–1/2 of the nerve thickness was trapped in the ligature. The wound was then closed with two to three skin sutures (4–0).

### Seahorse XF extracellular flux analyzer

At 1, 7, 14, and 28 days post-PSNL injury, mice were sacrificed by isoflurane overdose and cervical dislocation. Approximately 1.5 cm long lengths of sciatic nerves were immediately extracted and placed in ice cold XF base medium minimal DMEM at pH 7.4, supplemented with 5.5 mM glucose (Sigma-Aldrich), 0.5 mM sodium pyruvate (Invitrogen) and 1 mm glutaMAX (Invitrogen). Nerves were cut into three 3 mm segments using an acrylic V-shaped 1.0 mm slicer matrix (Zivic Instruments). Nerve segments were then cut in half longitudinally with a razor blade, and then further cut again along the cross-sectional axis twice to create 1 mm long hemi-sections. The pieces of a 3 mm long length of sciatic nerve were transferred into a single well of a XF96 cell culture microplate (Seahorse Bioscience), with 140 µl of cold supplemented XF base medium. Each nerve was tested in triplicate, with each 3 mm section making up one single triplicate. Various time points after nerve injury were tested, including contralateral (n = 27), ipsi day 1 (n = 5), ipsi day 7 (n = 12), ipsi day 14 (n = 5), and ipsi day 28 (n = 7). Oxygen consumption rates and extracellular acidification rates were measured with an XF 96 Analyzer (Seahorse Bioscience). An assay cycle of 2 min mix, 2 min wait, and 3 min measure protocol was repeated four times for baseline rates, and three times after each injection port. Injection ports contained: oligomycin A (20 µl, 12.5 µM), FCCP (20 µl, 12.5 µM), rotenone with antimycin A (20 µl total, both drugs 12.5 µM), and 2-deoxy-d-glucose (20 µl, 1 M).

### Statistics

All data is presented as mean ± SEM. Bonferonni’s multiple comparison test was performed post hoc to compare all measures to one another. The criterion for statistical significance was p < 0.05.

## Abbreviations

ASIC: acid sensitive ion channel
ATP: adenosine triphosphate
COX: cytochrome C oxidase
DMEM: Dulbecco’s modified eagle medium
FCCP: carbonyl cyanide *p*-trifluoromethoxyphenylhydrazone
HIF: hypoxia inducible factor
HIV: human immunodeficiency virus
NADH: nicotinamide adenine dinucleotide
NADPH: nicotinamide adenine dinucleotide phosphate
PSNL: partial sciatic nerve ligation
ROS: reactive oxygen species
TRPV1: transient receptor potential cation channel, subfamily V, member 1
